# En bloc vertebrectomy for the treatment of spinal lesions. Five years of experience in a single institution: a case series

**DOI:** 10.6061/clinics/2018/e95

**Published:** 2018-04-21

**Authors:** Alex Oliveira de Araujo, Douglas Kenji Narazaki, William Gemio Jacobsen Teixeira, Cesar Salge Ghilardi, Pedro Henrique Xavier Nabuco de Araujo, Antônio Eduardo Zerati, Raphael Martus Marcon, Alexandre Fogaça Cristante, Tarcísio Eloy Pessoa de Barros

**Affiliations:** I Divisao de Cirurgia da Coluna, Tumores da Coluna, Instituto de Ortopedia e Traumatologia (IOT), Hospital das Clinicas HCFMUSP, Faculdade de Medicina, Universidade de Sao Paulo, Sao Paulo, SP, BR; II Instituto do Cancer do Estado de Sao Paulo, Hospital das Clínicas HCFMUSP, Faculdade de Medicina da Universidade de Sao Paulo, Sao Paulo, SP, BR; III Divisao de Cirurgia da Coluna, Laboratorio de Investigacao Medica, Instituto de Ortopedia e Traumatologia (IOT), Hospital das Clinicas HCFMUSP, Faculdade de Medicina, Universidade de Sao Paulo, Sao Paulo, SP, BR

**Keywords:** Reconstructive Surgical Procedures, Spinal Neoplasms, Spine, Operative Surgical Procedures, Thoracic Surgery, Orthopedics

## Abstract

**OBJECTIVES:**

The objective of this study is to describe the experience of a Brazilian public university hospital regarding the treatment of metastatic or benign spine lesions with en bloc vertebrectomy of the thoracic and lumbar spines.

**METHODS:**

This study was a retrospective case series and included all medical records of patients with benign aggressive, primary malignant, or metastatic spine lesions who underwent en bloc vertebrectomy from 2010 to 2015.

**RESULTS:**

A total of 17 patients were included in the analysis. Most of them (71%) were indicated for surgery based on an oncologic resection for localized disease cure. Overall, 10 of the 17 patients (59%) underwent vertebrectomy via an isolated posterior approach using the technique described by Roy-Camille et al. and Tomita et al., while 7 patients (41%) underwent double approach surgeries. Of the 17 patients who underwent the en bloc resection, 8 are still alive and in the outpatient follow-up (47%), and almost all patients with metastatic lesions (8/9) died. The average survival time following the surgical procedure was 23.8 months. Considering the cases of metastatic lesions and the cases of localized disease (malignant or benign aggressive disease) separately, we observed an average survival time of 15 months and 47.6 months respectively.

**CONCLUSION:**

This study demonstrates and reinforces the reproducibility of the en bloc vertebrectomy technique described by Tomita et al.

## INTRODUCTION

The spine is the most common site of metastatic bone lesions in cancer patients. Approximately 70% of cancer patients will have metastases in the spine due to the dissemination of segmental arteries through the system or via the Batson venous plexus (a network of valveless veins) 1,2.

En bloc vertebrectomy, also called spondylectomy, has become an increasingly widespread procedure used for the treatment of neoplastic spine lesions. It consists of a resection of the entire tumor and one of more affected vertebrae with margins. The objective of this surgery is either cure or localized control of the disease (Enneking's principle of oncologic surgery). Several previous studies have demonstrated the superiority of vertebrectomies for localized control compared to intralesional tumor resection 3-5.

The evolution of spinal fixation methods in recent years has enabled the development of safer and improved methods of performing en bloc resections on the spine. Stener 6 was the pioneer in this type of resection and was followed by Roy-Camille et al., 7 and then by Tomita et al. 8 and Kawahara et al. 9.

A total vertebrectomy procedure results in complete loss of spinal continuity and stability. Thus, the best forms of spine stabilization and reconstruction seek maximum stability and have been widely studied and discussed. There are several strategies available including acute shortening of the spine, which was described by Kato et al. This approach has increased stabilization following vertebral reconstruction 10,11. The en bloc vertebrectomy has been used for patients with primary and metastatic spine lesions due to technical improvements and diffusion of this strategy 2,5.

The procedure has high morbidity-mortality and complication rates despite the technological advances. Previous studies have compared the risks and benefits of surgery that respects the oncologic margins, which was described by Enneking, and sacrificing important structures that cause serious functional problems. There are also smaller-scale surgeries that do not respect these criteria and are used in combination with adjuvant therapy 3. The principal adverse events associated with the technique include neurological deficit, postoperative infection, bleeding, failure of the synthesis material, cerebral spinal fluid fistula, and death. Additionally, adjuvant treatments with chemotherapy and radiotherapy can prolong patient recovery time and should also be considered 3,4,12.

Previous studies have focused on the survival implications and quality of life for patients who undergo the procedure due to the high number of associated complications. A retrospective case-control study by Colman et al. reported that the quality of life of 27 patients who underwent en bloc vertebrectomy was significantly worse than the general population in the United States 12. However, a study conducted in 2013 by Mazel et al. examined 25 patients who underwent partial or total vertebrectomy and observed the quality of life was satisfactory in both the medium and long terms compared with the general population regardless of the number of vertebrae resected 13.

There are a limited number of studies in the literature with samples of significant sizes due to the rarity of these tumors. The objective of this study is to report the experience with en bloc thoracic and lumbar vertebrectomies at the Instituto do Câncer do Estado de São Paulo over a five-year period. We also highlight the techniques used and the associated complications.

## METHODS

This study was a retrospective case series analysis conducted in a reference center for the treatment of cancer patients, which is part of the Brazilian public health system. The data were obtained from all the medical reports of patients treated from 2010 to 2015. We maintained the privacy of the personal information of all the patients included in the study. The protocol had the prior approval of the institutional ethics committee.

The inclusion criteria were patients with benign aggressive, primary malignant, or metastatic spine lesions who underwent en bloc vertebrectomy during the study period. All the surgeries were performed by the same surgeon, who is one of the authors (DN). All surgeries were performed at the same institution.

The following data were collected for all patients: age, sex, comorbidity, level of the lesion, type of surgical approach, surgical complications, survival, and classifications according to the Frankel (regarding neurological deficit at initial presentation) and Karnofsky (which classifies the patient in terms of the degree of their disabilities or functional deficiencies) scales. The modified Tokuhashi score was applied to the patients with malignant neoplasia. The SINS (Spinal Instability Neoplastic Score) classification system was used to assess all patients with tumor-related instability through six component scores (spine location, pain, lesion bone quality, radiographic alignment, vertebral body collapse, and posterolateral involvement of vertebrae).

We also used Enneking's tumor staging and the WBB classification (Weinstein, Boriani, Biagini), which describes the vertebral involvement according to layers. We used X-ray, magnetic resonance, and computed tomography images in addition to photographic images of the anatomical parts. In some cases, the images were taken intraoperatively for illustrative purposes.

A literature review was conducted in the PubMed database using the following search terms: vertebrectomy, classification system, total spondylectomy, spine tumors, en bloc resection, and complications.

The objectives of this case series report were to describe the experience of our institution with en bloc vertebrectomy, to show the profiles of the patients and the major complications associated with the procedure, and to present this information in the context of the current literature.

## RESULTS

A total of 17 patients underwent vertebrectomy procedures between 2010 and 2015 at the same institution. The average patient age was 44.5 years, and the median age was 52 years (range: 18-68 years). There were 9 (53%) male patients (Table 1). The patients had different types of tumors. There were 9 patients (53%) with metastatic tumors and 5 (29%) with benign aggressive tumors. There were also 3 patients (18%) with primary malignant tumors of the spine. The most common primary tumor was a kidney tumor (5 of 9, 55.5%) in patients with secondary lesions.

The Frankel scale showed 6 of the 17 patients (35%) scored Frankel E (no neurological deficit), 7 (41%) scored Frankel D, 1 scored Frankel C, and 3 scored Frankel B. The Karnofsky index results showed the following patient scores: 6 of the 16 patients (37.5%) scored 100 points, which indicates that the patient is completely able to perform normal activities and work activities, 2 (12.5%) scored 80 points, 2 (12.5%) scored 70 points, 2 (12.5%) scored 60 points, 1 (6.2%) scored 50 points, and the remaining 3 patients (18.8%) scored 40 points.

The Tokuhashi score applied to the 12 patients with malignant neoplasias. There were 4 patients (33.3%) who scored 0 to 8 points (average survival of 6 months) and 8 (66.7%) patients were in the range from 9 to 11 points (survival of 6 to 12 months).

The main reasons for indication of the vertebrectomies were the following: 12 of the 17 (71%) patients were indicated for oncologic resection for localized curing of the disease, 2 (11.5%) for compression of the cauda equina, 1 (6%) for medullary compression, and 2 (11.5%) for tumor recurrence.

The Enneking staging showed 5 of the 17 patients (29%) had benign aggressive tumors classified as type 3. The data indicate 9 of the 17 patients (53%) with malignant tumors had Enneking type 3 metastatic tumors. There were 3 patients (18%) with high grade malignant extracompartmental tumors without distant metastases, which are classified as Enneking type 2B 4.

The WBB scale showed 13 of the 17 patients (76%) had tumors that occupied the entire vertebral body and 5 tumors (38%) occupied only the body (areas 4-9). There were 8 tumors (62%) that occupied the entire vertebra (areas 1-12). The vertebra was affected on only one side in 4 of the 17 patients (24%). There were 3 tumors on the left (areas 1-6) and 1 on the right (areas 8-12). All the patients underwent en bloc vertebrectomy. The data showed 14 of the 17 tumors (82%) had invaded the epidural space (layer D).

The SINS score revealed 3 of the 17 patients (18%) had scores ranging from 0 - 6 (stable lesions), 13 (76%) had scores between 7 and 12 (lesions with undefined instability), and 1 (6%) had a score of between 13 and 18 (unstable lesion; Table 2).

**Figure 1:**
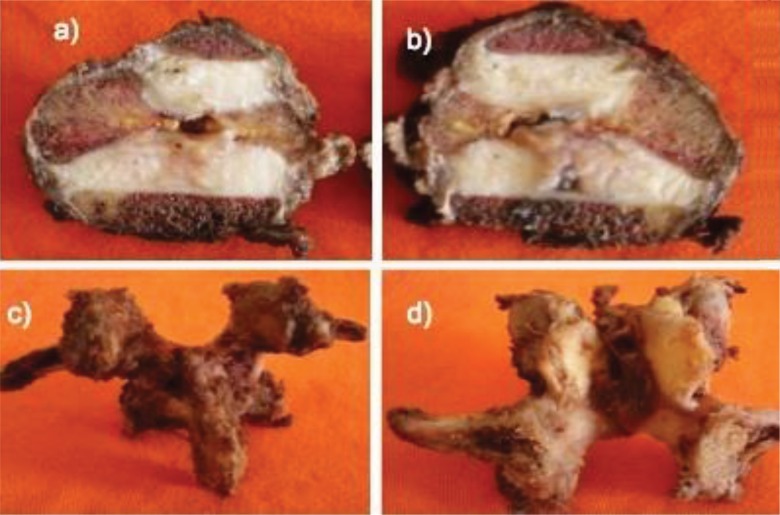
Vertebral specimen removed from a case of a giant cell tumor; a) and b) sagittal sections of L3; c) and d) posterior elements of L3.

The vertebrectomies were performed either by the single posterior approach or by the double approach, which was either anterior or lateral and posterior. There were 10 patients (59%) who underwent vertebrectomy via an isolated posterior approach using the technique described by Roy-Camille et al. 7 and Tomita et. al. (Figure 1) 8. The remaining 7 patients (41%) underwent double approach surgeries. In most cases, only one vertebra was resected en bloc. However, more than one vertebra was resected in four cases. A maximum of four vertebrae were resected en bloc in one patient. This case had vertebrae T10, T11, T12, and L1 removed. In another case, three vertebrae were resected (T12, L1, and L2) using the double approach. The levels operated on varied from T5 to L4 and in most cases where the resection of lumbar vertebrae was indicated we used the double approach for the procedure. In the majority of thoracic vertebrectomy cases we used the isolated posterior approach procedure. In addition, 4 of the 17 patients (23.5%) had undergone previous surgeries in another hospital before receiving treated in our hospital.

**Figure 2:**
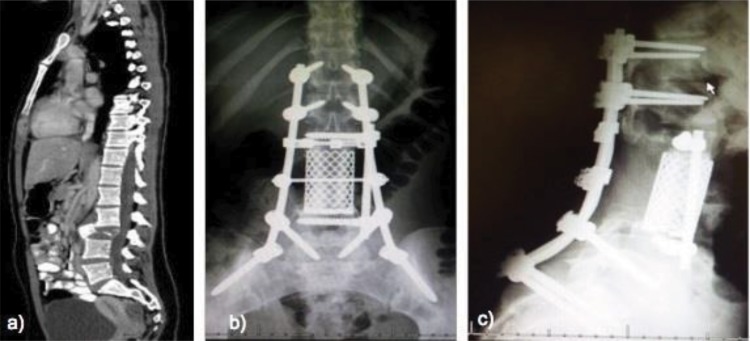
Computed tomography images of an 18-year-old patient with a giant cell tumor in L3. a) preoperative imaging b) and c) after en bloc vertebrectomy.

The anterior spine of each patient was reconstructed anteriorly with a titanium mesh cage (Harms cage) following the en bloc vertebrectomy by replacing the body and then posteriorly fixing two or three levels above and below the vertebrectomy with pedicle screws and rods (Figure 2). In one of the cases that had four vertebrae resected en bloc we used a heterologous graft from the femur of a cadaver for the reconstruction of the anterior spine. In this case, the graft was attached by an intramedullary rod in the humerus.

The average total time of the surgeries performed in two stages was 782.2 minutes. The surgeries performed in a single operation took an average of 508.7 minutes. The average estimated total bleeding was 5,125 ml for the two-stage surgeries and 2,333 ml for the single surgeries. The average hospitalization time was 25.4 days and there was no significant difference between the double approach and isolated posterior approach groups.

The data showed 13 of the 17 patients (76.5%) had intra- and postoperative complications and 6 of these 13 patients (46%) required new surgical approaches. There was an acute infection of the surgical site in 5 of the 17 cases (29%) and all of these patients underwent one or more debridement procedures in the operating room. In one case (6%), there was an intraoperative spleen lesion and the patient underwent a splenectomy during the same surgery. In another case (6%), the patient evolved pseudoarthrosis and failure of the synthesis material without evidence of infection. This patient underwent a new procedure to revise the synthesis material. There was one patient death on the day following the surgical procedure due to vasoplegic hemorrhagic shock (surgical complication).

Within the cohort of 17 patients who underwent en bloc resection there are 8 patients alive and receiving outpatient follow-up (47%). Of these 8 patients, 5 (62.5%) have benign aggressive lesions in the spine and 3 (37.5%) have other lesions. There were two patients with Ewing sarcoma and the other patient has metastatic hemangiopericytoma. Most patients with metastatic lesions died (8 out of 9 cases). The other death occurred in a patient with sarcoma. The average survival time following the surgical procedure was 23.8 months. The survival period ranged from patients who died during the first month to patients who survived for 70 months.

We evaluated the cases of metastatic lesions and the cases of localized disease (malignant and benign aggressive) separately. Our analysis showed the average survival time was 15 months for patients with metastatic lesions and of 47.6 months for patients with localized malignant or benign aggressive disease.

## DISCUSSION

Vertebrectomy is a highly complex procedure that has improved over the last several years. A limited number of centers worldwide perform this type of surgery due to the restricted indications. The learning curve is influenced by these two factors, and the level of complexity and rarity of this procedure means it is restricted to centers specializing in large-scale oncologic surgery. A multidisciplinary team and state-of-the-art technology are needed to achieve the best outcomes in the cases indicated.

Our institution specializes in cancer and was inaugurated in 2008. A combination of improvements in technology and an increase in the number of cases from both Brazil and other parts of South America has increased the number of large-scale surgeries. This study reports five years of experience with vertebrectomies at the ICESP (Cancer Institute of the state of São Paulo).

The indication of en bloc vertebrectomy depends on the following factors: age, comorbidities, clinical presentation, tumor type, distant metastases, performance, localized dissemination with invasion of important structures, and the number and degree of vertebrae involved. The patients were classified by different scores during the decision-making process to separate the cases into groups with respect to survival, instability, tumor aggressiveness, clinical performance, and location of vertebral involvement. In this study, we used the Frankel, Karnofsky, Enneking, Tokuhashi, SINS, and WBB classifications. The different scores from each of these classifications were used together with the individual case assessments to determine definitive indications for en bloc vertebrectomies.

Vertebrectomy is indicated for cases of metastatic and localized disease to control the disease either locally or systemically. In general, the patients who underwent en bloc resections were young adults (averaging 48 years of age) and demonstrated good clinical performance (average Karnofsky score of 75). We observed patients with systemic metastatic disease, and the average survival following the procedure was considered satisfactory at 15 months. The patients with metastatic disease had an expected survival of 6 to 12 months according to the Tokuhashi classification (between 0 and 11 points). However, these patients achieved an average survival of 15 months after the surgery. Most patients with metastatic lesions had radio- and chemo-resistant tumors, which can occur in kidney and thyroid neoplasias. These patients account for more than half of the cases. Metastatic lesions often reduce quality of life in cancer patients and are frequently associated with pathological fractures, spinal compression, and untreatable pain 2. Recent studies have shown the resection of metastases in selected cases can prolong survival or reduce local recurrence compared to intralesional resections 2,14-16.

The patients with localized disease survived for an average of 34 months and several patients remain in outpatient follow-up. In a recent retrospective study, Kato et al. published a series of 29 cases with more than 10 years of follow-up after en bloc vertebrectomy. The authors reported 19 cases had primary lesions (low-grade benign or malignant tumors) and 10 were metastatic lesions with a single metastasis 11. Schwab et al. reported the clinical results of 19 patients with spinal osteosarcoma and concluded the survival of the cases with aggressive lesions remained low. However, there was a tendency to opt for en bloc vertebrectomies because the survival is superior to intralesional resections 17.

The SINS score aims to identify spinal instability according to clinical and imaging criteria. A standalone analysis of the SINS score showed that most patients had lesions in the unknown stability category. This reinforces the concept that it is difficult to establish the degree of instability of tumor lesions 18.

The high complication rate encountered (76.5%) in our study is one of the factors that makes this surgery challenging. In a recent study, Mesfin et al. reported their experience with isolated posterior approach en bloc vertebrectomies over a period of 13 years. The authors highlighted complications such as injuries to the pleura, aorta, and vena cava, significant blood loss, failure of the synthesis material, and protrusion of the synthesis material that requires removal 1. In a case report published in 2014, Kawsar et al. described an anterior herniation of the spinal cord five years after resection in a patient with a giant cell tumor 19. The significant average amount of bleeding related to these procedures, the number of days in the hospital, and the number of associated complications are all factors that reinforce the need for a well-prepared multidisciplinary team for the successful management of these cases.

En bloc vertebrectomy is a surgical procedure that demands a high level of technical training both for the team of surgeons and for the hospital unit where it is performed. This study helps to demonstrate the reproducibility of the technique first described by Stener 6 and perfected by Roy-Camille et al. 7 and by Kawahara et al. 9. The long learning curve and the dependence on a specialized multidisciplinary approach are factors that limit greater diffusion of the procedure. However, additional experiences with progressively better results are being published worldwide and have contributed to the consolidation of the technique.

This study has a series of limitations such as the small patient sample, which is due to the restricted indications of this procedure. In addition, the small number of cases prevented a stratification of the different diseases into homogenous groups. Thus, we combined benign aggressive, primary malignant, and metastatic malignant diseases into a single group.

This is the first study published in South America that describes an experience with en bloc vertebrectomy as a treatment for primary and metastatic spine lesions. It is also one of the few studies conducted outside of the centers that pioneered the procedure.

## AUTHOR CONTRIBUTIONS

Araujo AO, Narazaki DK and Cristante AF designed the study, collected and interpreted the data and wrote the manuscript. Teixeira WG, Ghilardi CS, Araujo PH, Zerati AE and Marcon RM collected and interpreted the data, and critically revised the manuscript. Barros-Filho TE designed the study and critically revised the study. All authors revised the final version of the manuscript to be published and are responsible for the content.

## Figures and Tables

**Table 1:** Description of 17 patients submitted to en bloc vertebrectomy in a single institution in Brazil.

N	Age (years)	Sex	Tumor	Level	Approach	Survival (months)	Death
1	18	Female	Giant cell tumor	T9	Posterior	70	No
2	37	Male	Metastatic hemangiopericytoma	T12	Posterior	68	No
3	44	Female	Aggressive hemangioma	T5	Posterior	58	No
4	52	Female	Kidney	L4	Double	1	Yes
5	63	Female	Kidney	T9	Posterior	30	Yes
6	63	Male	Hemangioendothelioma	T11	Posterior	42	No
7	52	Female	Squamous cell carcinoma of unknown origin	L4	Posterior	26	Yes
8	53	Female	Kidney	L2	Double	4	Yes
9	63	Male	Kidney	T12-L1-L2	Double	1	Yes
10	68	Male	Esophagus	L3	Double	2	Yes
11	63	Female	Kidney	L3	Posterior	2	Yes
12	60	Male	Thyroid	T7-T8	Posterior	0	Yes
13	28	Male	Thoracic spine sarcoma	T5-T6	Posterior	9	Yes
14	19	Male	Ewing sarcoma	T5	Posterior	15	No
15	20	Male	Giant cell tumor	L4	Double	12	No
16	33	Male	Ewing sarcoma	T10-L1	Double	8	No
17	18	Female	Giant cell tumor	L3	Double	56	No

**Table 2:** Description of 17 cases submitted to en bloc vertebrectomy in one single institution in Brazil according to the histological type and classification scores.

Tumor	Frankel	Karnofsky	Enneking	Tokuhashi	WBB	SINS	Survival (months)
TGC	D	100	3	NA	1-9 D	8	70
Hemangioma	B	40	3	NA	1-2 D	8	58
TGC	E	100	3	NA	1-6 D	9	12
Hemangioendothelioma	C	60	3	NA	1-12 D	9	42
TGC	D	100	3	NA	4-9 D	12	56
Hemangiopericytoma	E	100	3	10	8-12 C	9	68
Kidney	D	60	3	10	4-9 D	8	1
Kidney	B	40	3	9	1-12 D	14	30
CEC	D	40	3	9	2-5 D	10	26
Kidney	E	70	3	9	1-12 D	11	4
Kidney	E	100	3	11	1-2 D	12	1
Esophagus	D	80	3	6	4-9 C	8	2
Kidney	E	100	3	10	4-9 D	5	2
Thyroid	E	80	3	11	1-5 D	6	0
Thoracic spine sarcoma	D	70	2B	7	1-12 D	11	9
Ewing sarcoma	B	50	2B	7	1-2 D	6	15
Ewing sarcoma	D	100	2B	8	1-12 C	10	8

WBB = Weinstein, Boriani, Biagini score; SINS = Spinal Instability Neoplastic Score.
